# Effect of light restriction on productive results and behavior of broiler chickens

**DOI:** 10.1016/j.psj.2023.103084

**Published:** 2023-09-01

**Authors:** F. Gratta, M. Bošković Cabrol, G. Xiccato, M. Birolo, F. Bordignon, A. Trocino

**Affiliations:** ⁎ Department of Agronomy, Food, Natural resources, Animals and Environment (DAFNAE), University of Padova, 35020 Legnaro, Padova, Italy; † Department of Comparative Biomedicine and Food Science (BCA), University of Padova, 35020 Legnaro, Padova, Italy

**Keywords:** genotype, sex, growth, meat quality, myopathy

## Abstract

The study aimed to evaluate the effect of light restriction (18L:6D vs. 14L:10D), genotype (A vs. B), and sex on performance, behavior, and meat quality, and the occurrence of wooden breast (**WB**) and white striping (**WS**) in broiler chickens. To this purpose 704 one-day-old chickens of 2 genotypes, half males and half females, were reared from hatching until slaughtering at 45 d of age in 32 collective pens (22 chickens per pen). Light restriction reduced growth rate and final live weight (**LW**), but improved feed conversion ratio (**FCR**) (*P* < 0.01) and reduced inactive behaviors of chickens (*P* < 0.001). Light restriction also reduced WS occurrence in breasts (89.5 to 64.6%; *P* < 0.001) and reduced meat shear force (2.64 to 2.20 kg/g; *P* < 0.05) and ether extract content (2.29 to 1.87%; *P* < 0.05). Regarding genotype, compared to genotype B, chickens of genotype A were heavier (3,242 g vs. 3,124 g; *P* < 0.01) with higher cold carcass weight and *Pectoralis major* muscle yield (12.9 vs. 12.0%; *P* < 0.001) and a higher FCR (1.63 vs. 1.61; *P* < 0.01). Finally, females had lower final LW (2,852 g vs. 3,513 g) and higher FCR (1.64 vs. 1.59) than males (*P* < 0.001), but a higher proportion of breast and *P. major* (*P* < 0.001), lower cooking losses (*P* < 0.001) and shear force (*P* < 0.01), and higher protein content (21.6 vs. 20.7%; *P* < 0.001). In conclusion, light restriction depressed growth, but was effective in decreasing WS occurrence and improved feed conversion. The decrease in inactive behaviors (sitting/laying) of light-restricted chickens can be positively considered in view of animal welfare.

## INTRODUCTION

In recent decades, genetic selection for growth rate and feed efficiency in broiler chickens increased productivity but led to the development of some metabolic disorders and degenerations, including breast myopathies (Petracci et al., 2015). White striping (**WS**) and wooden breast (**WB**) are the myopathies mostly observed, characterized by different macroscopic aspects and common histological features (Kuttappan et al., 2013a; Sihvo et al., 2014). While producing a sufficient amount of broiler meat is a priority, producing high-quality meat is also an important goal (Caldas-Cueva and Owens, 2020) with special reference to appearance, water-holding capacity (**WHC**), color, and texture that influence sensory properties and eating quality, and, thus consumers’ acceptance. The aforementioned muscle abnormalities may worsen meat quality, forcing producers to process or destroy meat due to altered aspects and technological properties (Petracci et al., 2014). Carcass downgrades or condemnation because of myopathies has been estimated to cause daily losses of up to U$ 70,632 per d in Brazil (Zanetti et al., 2018) and more than >U$ 1 billion per year in the United States (Barbut, 2020). Additionally, myopathies may affect bird behavior and have potential welfare consequences for chickens (Kawasaki et al., 2016; Norring et al., 2019; Cônsolo et al., 2022).

The etiology of these muscle abnormalities has not been fully elucidated, although literature data imply that heritability has a negligible role (Bailey et al., 2020) and environmental and/or management factors contribute more than 90% of the variance of WB occurrence and more than 65% of the variance of WS (Bailey et al., 2015). Increased LW is a risk factor for both WB and WS occurrence (Che et al., 2022), whereas the association between high-breast yield and WB and WS development has also been reported (Lake et al., 2020; Bordignon et al., 2022). Some authors (Trocino et al., 2015; Pascual et al., 2020) found that sex plays a role in the occurrence and severity of the different myopathies.

Until now, different strategies—like feed restriction (Trocino et al., 2015; Gratta et al., 2019), supplementation with different dietary additives (Estevez and Petracci, 2019; Pascual et al., 2020; Khan et al., 2021; Souza et al., 2021), different stocking densities (Cônsolo et al., 2022), genetic selection and use of slow-growing genotypes (Santos et al., 2021), reduction of slaughter age (Kuttappan et al., 2017)—have been tested to mitigate WS and WB occurrences, most of them achieving positive results through slowing down the growth rate, decreasing final live weight (**LW**) and/or breast yield. A decrease in LW due to a restriction of lighting hours has been previously described (Classen et al., 1991; Classen, 2004); its effect on the occurrence and severity of WS or WB has not been evaluated whereas changes of broiler behavior and welfare are likely to occur (Olanrewaju at al., 2019).

Thus, the present study aimed to evaluate whether a light restriction may affect productive performance, meat quality, and the occurrence and severity of myopathies, besides the behavior, in both sexes of 2 widely used fast-growing chicken genotypes.

## MATERIALS AND METHODS

### Ethical Statement

All procedures used in the present experiment were approved by the Ethical Committee for Animal Experimentation of the University of Padova (project number: 17/2016; Prot. n. 154392, 10/05/2016). All animals were handled in respect to the principles stated by the EC Directive 2010/63/EU regarding the protection of animals used for experimental and other scientific purposes. The researchers involved in animal handling were either animal specialists (PhD or MSc in Animal Sciences) and/or veterinary practitioners.

### Animals, Housing, and Management

This experiment was conducted at the poultry house of the Experimental Farm of the University of Padova (Legnaro, Padova, Italy), equipped with cooling system, forced ventilation, radiant heating, and controlled light systems, between the months of May to June, after a 6-mo downtime. A total of 704 one-day-old fast-growing commercial crossbred broiler chickens, half males and half females, half belonging to the genotype A and half to genotype B, were delivered by an authorized truck at the experimental farm in compliance with Council Regulation (EC) No 1/2005 to the experimental facilities. Chicks were sexed and vaccinated against Marek's disease, Infectious Bronchitis, and Newcastle disease at the hatchery.

At their arrival, 22 chicks per pen were housed in 32 pens (2.2 m^2^; 125 cm wide × 177 cm large × 120 cm height) in the 2 twin rooms of the poultry house (16 pens per room). The 32 pens were allocated to 8 experimental groups, that is, 2 genotypes × 2 sexes × 2 photoperiods (18 h vs. 14 h of light during 24 h). In details, during the first 24 h of the trial, all chicks were subjected to the same photoperiod schedule of 24L:0D. From the second day of age, the hours of lightness were gradually reduced until reaching 18L:6D at 9 d of age. From that moment onward in 1 room the 18L:6D photoperiod was maintained; in the other room the light period was reduced to 16 h (at 10 d of age) and 14 h (at 11 d of age) and then the 14L:10D photoperiod was maintained until the end of the trial.

All pens were equipped with an automatic circular drinker (diameter: 39 cm) and a circular feeder (diameter: 37 cm). The concrete floor was covered with wood shavings litter (height 5 cm, 2.5 kg/m^2^). All birds received the same commercial diets (Table 1), that is, the starter diet from housing to 13 d; the first grower diet from 14 d to 24 d of age; the second grower diet from 25 d to 36 d of age; and the finisher diet from 37 d of age until slaughtering at 45 d. Feed and water were provided ad libitum during the entire experiment.

**Table 1:** Calculated chemical composition (% as fed) of the commercial diets fed during the trial to broiler chickens.

Diet	Starter^1^	First grower^1^	Second grower^2^	Finisher^2^
Period of administration	1–13 d	14–24 d	25–36 d	37–45 d
Dry matter (%)				
Crude protein (%)	23.00	21.80	20.60	18.30
Ether extract (%)	6.50	7.90	8.10	8.00
Crude fiber (%)	2.60	2.50	2.50	2.40
Ash (%)	6.00	5.50	5.20	4.90
Lysine (%)	1.47	1.38	1.29	1.04
Methionine (%)	0.63	0.54	0.50	0.42
Calcium (%)	0.90	0.82	0.77	0.75
Phosphorous (%)	0.70	0.67	0.64	0.60
Sodium (%)	0.18	0.16	0.16	0.16

All diets contained: corn, soybean meal, animal fat, dicalcium phosphate, calcium carbonate, sodium chloride.

1 Premix provided per kg of feed: vit. A, 13,000 IU; vit. D3, 4,500 IU; vit. E acetate, 45 mg; iron carbonate (Fe): 90 mg; calcium iodate anhydrous (I): 2.70 mg; cupric sulfate (Cu): 35 mg; manganese oxide (Mn): 150 mg; zinc oxide (Zn): 110 mg; sodium selenite (Se): 0.40 mg; methionine hydroxy analogue: 3,600 mg; 6-phytase (EC 3.1.3.26): 250 OTU; serine protease (EC 3.4.21): 15.000 PROT; Narasin: 50 mg; Nicarzabin: 50 mg.

2 Premix provided per kg of feed: vit. A, 10,500 IU; vit. D3, 3,600 IU; vit. E acetate, 36 mg; iron carbonate (Fe): 72 mg; calcium iodate anhydrous (I): 2.15 mg; cupric sulfate (Cu): 28 mg; manganese oxide (Mn): 120 mg; zinc oxide (Zn): 90 mg; sodium selenite (Se): 0.30 mg; methionine hydroxy analogue: 3,600 mg; 6-phytase (EC 3.1.3.26): 250 OTU; serine protease (EC 3.4.21): 15,000 PROT; Narasin: 70 mg.

### In Vivo Recordings

Chicks were individually weighed on the day of their arrival, identified by a labeled plastic band at the leg, and weighed once per week to measure LW, besides promptly identifying any health problem. The pen feed intake and mortality were daily recorded.

At 11, 25, and 39 d of age, the behavior of chickens during 24 h was video recorded. To this purpose, infrared cameras (V700-20 Atlantis, ATL S.r.l., Pogliano Milanese, MI, Italy) were attached to the fences of the 16 pens and data were stored on hard drive by a digital video recorder (H.264 DVR-16 channels, RDS CCTV s.r.l., Montesilvano, PE, Italy). Then, the number of chickens per pen performing predetermined and mutually exclusive behaviors was scored every 30 min by scanning 10 consecutive seconds of video (Trocino et al., 2020). The following behaviors were selected based on Nielsen et al. (2011): feeding (chickens pecking in feeders); drinking (chickens at drinkers); standing up; sitting/lying down; walking (walking or running with no other discernible activity); pecking the floor (including the litter); pecking other bird (any body part); pecking their own tail; aggression; dust bathing; comfort (preening, scratching, or wing stretching).

### Commercial Slaughtering and Carcass and Meat Quality Recordings

At 45 d of age, after 7 h of feed and 4 h of water withdrawal, all chickens were slaughtered in a commercial slaughterhouse according to standard procedures. Loading took approximately 1 h; transport from the experimental facilities to the commercial slaughterhouse took approximately 15 min; and lairage in the transport cages under a shed before slaughtering took approximately 3 h. Ready-to-cook carcasses were recovered and individually weighed to measure the slaughter dressing percentage after 2 h of refrigeration at 2°C.

Among those carcasses, 192 ones (6 per pen) were selected, as representative in terms of average bird LW and variability of the corresponding pens, and submitted to gross examination for the occurrence and severity of WS and WB on *Pectoralis major* muscle according to the criteria proposed by Kuttappan et al. (2012) and Sihvo et al. (2014). Afterward, half of the carcasses (96 carcasses) were transported to the department laboratories to be stored at 2°C before carcass and meat quality analyses.

Twenty-four hours after slaughtering, carcasses were dissected in major parts (breast, wings, thighs, and drumstick). *Pectoralis major* muscles were separated from the breasts and the pH was measured in triplicate on their ventral side using a pH meter (Basic 20, Crison Instruments Sa, Carpi, Italy) equipped with a specific electrode (cat. 5232, Crison Instruments Sa). Color indexes were measured on 3 different sites on the ventral surface of the muscle using a Minolta CM-508 C spectrophotometer (Minolta Corp., Ramsey, NJ). Thereafter, 1 parallelepiped meat portion (8 cm × 4 cm × 3 cm) was separated from the cranial side of *P. major*, parallel to muscle fibers directions, packaged under vacuum and stored at −18°C until measuring thawing and cooking losses, and shear force. The remaining meat was freeze-dried, reground, and used to determine proximate composition, that is, dry matter (934.01), ash (967.05), crude protein (2001.11), and ether extract (991.36) contents (AOAC, 2000).

Thawing and cooking losses were measured according to Petracci and Baéza (2011). Briefly, after thawing at 4°C for 24 h, the meat portion was unpackaged, gently wiped with paper and weighed to determine thawing losses, then it was packaged under vacuum again and cooked in a water bath to an internal temperature of 80°C. The cooked meat samples were cooled in 40 min at room temperature, gently wiped with paper and reweighed to determine cooking losses. Then, a parallelepiped meat portion (4 cm × 2 cm × 1 cm) was obtained from the cooked sample to measure the shear force using a LS5 dynamometer (Lloyd Instruments Ltd., Bognor Regis, UK) with the Allo-Kramer (10 blades) probe (load cell: 500 kg; distance between the blades: 5 mm; blade thickness: 2 mm; cutting speed: 250 mm/min) (Mudalal et al., 2015).

### Statistical Analysis

Individual data of LW, daily weight gain (**DWG**), slaughter yield, and carcass and meat traits were analyzed by ANOVA with photoperiod, genotype, and sex as the main factors of variability with interactions, and with pen as a random effect using the PROC MIXED of SAS software (SAS Institute, 2013). Cage data of daily feed intake (**DFI**) and feed conversion rate (**FCR**) were analyzed by ANOVA with the same main factors of variability by the PROC GLM of SAS. When due, the Bonferroni *t* test was used to compare least squares means. The occurrence of myopathies at *P. major* was analyzed by PROC CATMOD of SAS.

Behavioral data (as a percentage of animals performing a behavior in each pen per scan) were subjected to analysis of variance by using a mixed model and the PROC GLIMMIX of SAS, with photoperiod, genotype, sex, and animal age, and their interactions as fixed effects and hour as a random effect. A Poisson distribution was assumed for these data. Results related to significant interactions among factors for LW, DWG, and DFI (Table S1) and for behavior (Table S2, Figures S1–S3) are available as Supplementary materials and not discussed in the manuscript.

## RESULTS

### Growth Performance and Slaughtering Results

Chickens reared with less light hours showed a lower final LW compared to the other chickens (3,236–3,130 g) which corresponded to a lower DWG (−3.3%) and DFI (−4.3%) (*P* < 0.001) in the whole experimental period (Tables 2 and 3). Namely, differences in DFI were observed after 10 d of age, when lighting regime changed between the 2 groups. At slaughtering, differences in carcass weights were consistent with changes in final LW: chickens kept with less light hours had lighter carcasses that exhibited higher proportions of wings and thighs at dissection compared to the other chickens (*P* < 0.01) (Table 4).

**Table 2:** Effect of photoperiod, genotype, and sex on live weight (LW) and daily weight gain (DWG) (LS means) of broiler chickens (individual data) from hatching until slaughtering at 45 d of age.

	Photoperiod (P)		Genotype (G)		Sex (S)		*P* value						MSE
Variables	18L:6D	14L:10D	A	B	F	M	P	G	S	P × G	G × S	P × S	
Chickens (n)	330	330	329	331	330	330							
LW (g)													
D 1	48.8	48.4	51.6	45.6	47.6	49.5	0.60	<0.001	<0.01	0.16	<0.01	0.92	1.9
D 10	269	270	284	255	259	281	0.68	<0.001	<0.001	0.83	<0.001	0.60	8
D 17	645	617	650	612	598	664	<0.001	<0.001	<0.001	0.83	<0.001	0.79	20
D 24	1195	1122	1192	1125	1079	1238	<0.001	<0.001	<0.001	0.26	<0.01	0.44	41
D 31	1882	1785	1886	1781	1663	2003	<0.001	<0.001	<0.001	0.10	<0.05	0.63	67
D 38	2642	2502	2631	2512	2315	2829	<0.001	<0.001	<0.001	0.09	0.10	0.47	80
D 45	3236	3130	3242	3124	2852	3513	<0.01	<0.001	<0.001	0.67	0.09	0.98	88
DWG (g/d)													
D 1–10	24.5	24.7	25.9	23.3	23.5	25.7	0.59	<0.001	<0.001	0.95	<0.001	0.58	0.9
D 10–17	53.6	49.5	52.2	51.0	48.4	54.8	<0.001	0.10	<0.001	0.87	<0.01	0.93	1.9
D 17–24	78.6	72.1	77.5	73.2	68.7	82.0	<0.001	<0.01	<0.001	0.31	0.10	0.26	3.3
D 24–31	98.2	94.7	99.1	93.8	83.5	109	<0.05	<0.01	<0.001	0.24	0.32	0.98	4.5
D 31–38	109	102	106	104	93.0	118	<0.001	0.12	<0.001	0.57	0.30	0.31	3.6
D 38–45	91.4	96.7	93.9	94.1	82.7	105	<0.01	0.90	<0.001	0.09	0.53	0.08	4.1
Overall	72.4	70.0	72.5	70.0	63.7	78.7	<0.01	<0.01	<0.001	0.75	0.11	0.98	2.0

MSE: root mean square error.

**Table 3:** Effect of photoperiod, genotype, and sex on daily feed intake (DFI) and feed conversion ratio (FCR) (LS means) of broiler chickens (pen data) from hatching until slaughtering at 45 d of age.

	Photoperiod (P)		Genotype (G)		Sex (S)		*P* value						MSE
Variables	18L:6D	14L:10D	A	B	F	M	P	G	S	P × G	G × S	P × S	
Pens (n)	16	16	16	16	16	16							
DFI (g/d)													
D 1–10	27.7	27.5	29.2	26.0	26.5	28.7	0.60	<0.001	<0.001	0.23	<0.01	0.26	1.3
D 10–17	68.1	62.8	66.2	64.7	62.7	68.2	<0.001	0.16	<0.001	0.81	<0.01	0.86	2.7
D 17–24	110	103	111	102	99	114	<0.001	<0.001	<0.001	0.24	<0.05	0.57	4
D 24–31	143	137	145	135	125	155	<0.01	<0.001	<0.001	0.53	0.12	0.41	6
D 31–38	174	168	175	167	153	190	<0.001	<0.001	<0.001	0.94	0.99	0.67	5
D 38–45	181	177	180	178	162	195	<0.05	0.31	<0.001	0.22	0.43	0.68	6
Overall	117	112	118	112	105	125	<0.001	<0.001	<0.001	0.50	0.10	0.83	4
FCR													
D 1–10	1.13	1.12	1.13	1.12	1.13	1.12	0.25	0.46	0.37	0.10	0.08	0.34	0.04
D 10–17	1.27	1.27	1.27	1.27	1.30	1.24	0.78	0.93	<0.01	0.98	0.67	0.81	0.05
D 17–24	1.41	1.43	1.44	1.40	1.44	1.39	<0.05	<0.01	<0.001	0.70	0.25	0.38	0.03
D 24–31	1.46	1.45	1.47	1.45	1.50	1.42	0.35	<0.05	<0.01	0.09	0.21	0.10	0.02
D 31–38	1.61	1.64	1.65	1.60	1.64	1.61	<0.05	<0.01	<0.05	0.66	0.08	0.11	0.04
D 38–45	1.99	1.84	1.93	1.90	1.97	1.86	<0.001	0.29	<0.001	0.12	0.78	0.11	0.06
Overall	1.62	1.61	1.63	1.61	1.64	1.59	<0.01	<0.01	<0.001	0.13	0.43	0.57	0.02

MSE: root mean square error.

**Table 4:** Effect of photoperiod, genotype, and sex on carcass weight, yield and main cuts proportion (LS means) of broiler chickens slaughtered at 45 d of age.

	Photoperiod (P)		Genotype (G)		Sex (S)		*P* value						MSE
Variables	18L:6D	14L:10D	A	B	F	M	P	G	S	P × G	G × S	P × S	
Chickens (n)	48	48	48	48	48	48							
Cold carcasses (g)	2335	2107	2279	2162	1967	2474	<0.001	<0.001	<0.001	0.84	0.21	0.87	164
Dressing percentage (%)	73.8	73.5	73.6	73.7	73.2	74.1	0.31	0.62	<0.01	0.97	0.21	0.68	1.50
Breast yield (%)^1^	40.0	39.8	41.0	38.8	40.9	39.0	0.72	<0.001	<0.001	0.29	0.21	<0.05	2.1
*P. major* (%)	12.6	12.3	12.9	12.0	12.8	12.1	0.32	<0.001	<0.001	0.30	0.30	<0.05	0.9
Wings (%)	9.97	10.6	10.1	10.5	10.3	10.3	<0.01	<0.001	0.93	0.23	0.82	0.60	0.5
Thighs (%)	14.8	15.4	14.8	15.4	15.0	15.3	<0.01	<0.05	0.14	0.62	0.41	0.87	1.0
Drumsticks (%)	13.2	13.2	13.0	13.4	12.9	13.5	0.95	<0.01	<0.001	0.24	0.90	0.45	0.7

MSE: root mean square error. Significant probability of interaction photoperiod × sex: breast yield, 41.6 and 38.8% in female and male chickens at 18L:6D; 40.7 and 39.8% in female and male chickens at 14L:10D; P. *major*, 13.3 and 12.3% in female and male chickens at 18L:6D; 12.9 and 12.6% in female and male chickens at 14L:10D.

1 With bone and skin.

As for genotypes, differences were significant since the hatching day, with chicks of genotype A heavier than those of genotype B (+13.1%, *P* < 0.001). The former chickens showed a higher DWG and DFI during the whole trial for which the difference in LW at the end of the trial was still significant (+3.8%; *P* < 0.001). On the other hand, in the whole period, FCR was significantly higher for genotype A compared to genotype B (1.63 vs. 1.61; *P* < 0.01) (Table 3). At slaughtering and dissection, chickens of genotype A had higher cold carcasses weight and greater breast and *P. major* yields (*P* < 0.001), but a lower proportion of wings (*P* < 0.001), drumsticks (*P* < 0.01), and thighs (*P* < 0.05) in comparison with chickens of genotype B (Table 4).

As for sex, females were lighter than males since the hatching day (47.6 g vs. 49.5 g; *P* < 0.01) and until slaughtering (2,852 g vs. 3,513 g; *P* < 0.001) which corresponded to a lower DWG from the first (−8.6%; *P* <0.001) until the last week of the trial (−21.2%; *P* < 0.001) (Table 2). Besides, a lower DFI (−19.1; *P* < 0.001) and a higher FCR (1.64 vs. 1.59; *P* < 0.001) were recorded in the whole trial (Table 3). At dissection, females showed lower dressing percentage (*P* < 0.01) and drumstick proportions (*P* < 0.01), but higher breast and *P. major* yields (*P* < 0.001) compared to males (Table 4).

Significant interactions were recorded between genotype and sex for LW until 31 d of age, whereas differences for DWG and DFI were recorded until 24 d of age (Tables 2 and 3). In details, differences between males and females of genotype A were significant only at 17 d of age (Table S2). At slaughtering (Table 4), a significant interaction photoperiod × sex (*P* < 0.05) was measured on breast yield and *P. major* yield.

### Occurrence of Myopathies and Meat Quality

Chickens reared with 14 h of light showed a lower occurrence of WS breasts (64.6 vs. 89.5%; *P* < 0.001) and severe WS breasts (18.8 vs. 37.9%; *P* < 0.01) compared to chickens kept with 18 h of light (Table 5). The meat of the former chickens exhibited a lower shear force (2.20 kg/g vs. 2.64 kg/g; *P* < 0.05), besides higher water (75.7 vs. 75.3%; *P* < 0.05) and lower ether extract (1.87 vs. 2.29%; *P* < 0.05) contents (Table 6).

**Table 5:** Effect of photoperiod, genotype, and sex on WS and WB occurrence (means) in broiler chickens slaughtered at 45 d of age.

	Photoperiod (P)		Genotype (G)		Sex (S)		*P* value					
Variables	18L:6D	14L:10D	A	B	F	M	P	G	S	G × P	G × S	P × S
Chickens (n)	96	96	96	96	96	96						
White striping %	89.5	64.6	76.0	77.9	70.5	83.3	<0.001	0.80	0.26	0.52	<0.05	<0.05
1 (mild)	51.6	45.8	47.9	49.5	44.2	53.1	0.42	0.87	0.21	0.80	0.10	0.09
2 (severe)	37.9	18.8	28.1	28.4	26.3	30.2	<0.01	0.76	0.42	0.47	0.27	0.35
Wooden breast %	8.33	4.17	6.25	6.25	3.13	9.38	0.31	0.75	0.11	0.99	0.50	0.98

Significant probability of interaction genotype × sex: White striping, 75.0 and 77.1% in female and male chickens of genotype A; 66.4 and 89.6% in female and male chickens of genotype B. Significant probability of interaction photoperiod × sex: White striping, 91.4 and 87.5% in female and male chickens kept at 18L:6D; 50.0 and 79.2% in female and male chickens kept at 14L:10D.

**Table 6:** Effect of photoperiod, genotype, and sex on meat quality traits and chemical composition (LS means) of *pectoralis major* muscle of broiler chickens slaughtered at 45 d of age.

	Photoperiod (P)		Genotype (G)		Sex (S)		*P* value						MSE
Variables	18L:6D	14L:10D	A	B	F	M	P	G	S	P × G	G × S	P × S	
Chickens (n)	48	48	48	48	48	48							
pH	5.94	5.95	5.94	5.95	5.92	5.98	0.84	0.32	<0.01	0.19	0.48	0.94	0.11
L*	46.2	46.0	45.9	46.3	46.3	46.0	0.72	0.34	0.46	<0.01	0.56	0.10	2.0
a*	0.02	−0.18	−0.08	−0.08	−0.02	−0.15	0.08	1.00	0.25	0.10	0.11	0.77	0.54
b*	15.6	15.7	15.6	15.8	15.7	15.6	0.89	0.65	0.77	0.11	0.22	0.43	2.1
Thawing loss %	6.54	6.31	6.44	6.40	6.34	6.51	0.76	0.92	0.68	0.24	0.90	0.30	1.89
Cooking loss %	28.3	28.0	27.0	29.3	26.4	29.9	0.73	<0.001	<0.001	0.88	0.77	0.59	3.2
Shear force kg/g	2.64	2.20	2.29	2.55	2.26	2.58	<0.05	<0.01	<0.01	0.55	0.71	0.29	0.46
Chemical composition (%)													
Water	75.3	75.7	75.5	75.5	75.1	75.9	<0.05	0.97	<0.001	0.95	0.88	0.50	0.8
Ash	1.09	1.06	1.08	1.07	1.08	1.07	<0.05	0.09	<0.05	0.22	0.06	0.77	0.03
Crude protein	21.2	21.1	21.3	21.0	21.6	20.7	0.47	0.06	<0.001	0.75	0.74	0.24	0.9
Ether extract	2.29	1.87	1.94	2.22	1.94	2.22	<0.05	0.06	0.06	0.65	0.42	0.56	0.72

MSE: root mean square error. Significant probability of interaction photoperiod × genotype: Lightness L*, 46.6 and 45.8 in chickens of genotype A and B kept at 18L:6D; 45.3 and 46.8 in chickens of genotype A and genotype B kept at 14L:10D.

Genotype did not affect the occurrence of myopathies, whereas lower cooking losses (27.0 vs. 29.3%; *P* < 0.001) and shear force values (2.29 kg/g vs. 2.55 kg/g; *P* < 0.01) were measured on the breast meat in chickens of genotype A compared to chickens of genotype B.

Finally, differences in WS and WB occurrence between chickens of the 2 sexes were only numerical (*P* > 0.05), whereas females showed lower pH (5.92 vs. 5.98; *P* < 0.01), cooking losses (26.4 vs. 29.9%; *P* < 0.001), and shear force (2.26 kg/g vs. 2.58 kg/g; *P* < 0.01) at the *P. major* muscle compared to males (Table 6). Moreover, females exhibited a lower water (75.1 vs. 75.9%; *P* <0.001) and higher crude protein contents (21.6 vs. 20.7%; *P* <0.001) of meat compared to males.

### Behavioral Recordings

The reduction of lighting hours increased the rate of chickens observed drinking (7.45 to 8.98%; *P* < 0.01) and, importantly, reduced the rate of chickens sitting or lying down (49.7 to 44.2%; *P* < 0.001) (Table 7). As for differences between genotypes, namely, differences were a few, that is, the rate of chickens pecking the floor (11.7 vs. 13.1%; *P* = 0.05) was lower in genotype A compared to genotype B. Finally, sex did not affect the chicken behavior.

**Table 7:** Effect of photoperiod, genotype, sex, and age on the behavior of broiler chickens (percentage of chickens showing a behavior per pen) (means ± SEM).

	Photoperiod (P)		Genetic line (G)		Sex (S)		Age (A)			*P* value			
Behaviors	18L	14L	A	B	F	M	11 d	25 d	39 d	P	G	S	A
Feeding	12.8 ± 0.35	13.0 ± 0.42	13.4 ± 0.39	12.4 ± 0.39	13.3 ± 0.39	12.4 ± 0.37	18.6^b^ ± 0.50	10.6^a^ ± 0.39	9.40^a^ ± 0.38	0.68	0.10	0.17	<0.001
Drinking	7.45 ± 0.22	8.98 ± 0.29	7.66 ± 0.24	8.58 ± 0.24	7.67 ± 0.24	8.57 ± 0.26	8.44^b^ ± 0.27	8.77^b^ ± 0.33	7.15^a^ ± 0.30	<0.01	0.07	0.10	<0.01
Standing	4.36 ± 0.21	5.25 ± 0.24	4.90 ± 0.22	4.60 ± 0.22	4.86 ± 0.21	4.64 ± 0.23	4.05^a^ ± 0.23	5.73^b^ ± 0.33	4.46^a^ ± 0.25	0.09	0.41	0.42	<0.01
Sitting/lying	49.7 ± 0.52	44.2 ± 0.63	48.0 ± 0.58	46.6 ± 0.58	46.9 ± 0.57	47.7 ± 0.58	40.0 a ± 0.68	46.4^b^ ± 0.66	55.5^c^ ± 0.60	<0.001	0.15	0.26	<0.001
Walking	3.78 ± 0.18	4.33 ± 0.22	4.19 ± 0.20	3.89 ± 0.20	4.27 ± 0.20	3.77 ± 0.19	6.26^c^ ± 0.29	4.03^b^ ± 0.22	1.76^a^ ± 0.14	0.11	0.95	0.33	<0.001
Pecking floor	11.7 ± 0.27	13.3 ± 0.32	11.7 ± 0.28	13.1 ± 0.30	12.3 ± 0.29	12.5 ± 0.29	12.9 ± 0.37	13.3 ± 0.37	10.9 ± 0.31	0.08	0.05	0.81	<0.001
Pecking other birds	1.39 ± 0.09	1.37 ± 0.10	1.31 ± 0.09	1.44 ± 0.10	1.32 ± 0.09	1.43 ± 0.10	1.33 ± 0.11	1.46 ± 0.11	1.35 ± 0.11	0.91	0.41	0.37	0.19
Aggressiveness	0.33 ± 0.06	0.45 ± 0.08	0.35 ± 0.06	0.42 ± 0.06	0.29± 0.06	0.48 ± 0.08	0.62 ± 0.11	0.30 ± 0.08	0.24 ± 0.06	0.17	0.52	0.64	0.27
Pecking own tail	0.15 ± 0.03	0.09 ± 0.02	0.13 ± 0.03	0.12 ± 0.03	0.15 ± 0.03	0.10 ± 0.02	0.18^b^ ± 0.04	0.18^b^ ± 0.04	0.02^a^ ± 0.01	0.13	0.88	0.17	<0.001
Dustbathing	0.43 ± 0.05	0.44 ± 0.06	0.40 ± 0.05	0.46 ± 0.05	0.46 ± 0.05	0.39 ± 0.05	0.72 ± 0.09	0.36 ± 0.06	0.21 ± 0.04	0.44	0.85	0.65	0.48
Comfort	7.95 ± 0.19	8.66 ± 0.21	7.99 ± 0.20	8.54 ± 0.20	8.49 ± 0.21	8.03 ± 0.19	6.95^a^ ± 0.23	8.89^b^ ± 0.24	8.95^b^ ± 0.26	0.20	0.37	0.35	<0.001

a,b,c Means with different superscript letters are different (*P* < 0.05).

From the first to the last recording (11 d to 39 d of age), the rate of chickens feeding decreased (18.6 to 9.40%; *P* < 0.001). Chickens at drinkers remained stable (8.44 to 8.77%) until 25 d and then decreased (*P* < 0.01) to 7.15% by the last day of observation (39 d). Standing birds increased from 4.05 to 5.73% from 11 d to 25 d and then decreased to 4.46% at 39 d (*P* < 0.01), whereas those sitting or lying increased (40.0 to 55.5%) from the first observation (11 d) until the last one (39 d). Thus, walking birds definitely declined with age (6.26 to 1.77%; *P* < 0.001). The birds pecking the floor decreased (*P* < 0.001) over time. Finally, comfort behaviors were exhibited by 6.95% of chickens at 11 d of age and then increased to 8.9% from 25 d onward (*P* < 0.001).

## DISCUSSION

### Effect of Light Restriction

Being diurnal animals, birds are sensitive to changes in light intensity and duration of photoperiod (Olanrewaju et al., 2006; Rault et al., 2017) where light plays an important role in many regulatory functions affecting voluntary activity and physiology (Sanotra and Weeks, 2004; Meluzzi and Sirri, 2009). Long periods of darkness may limit the growth rate by preventing the regular access to feed and may be an important factor for broiler health (Classen et al., 1991). Olanrewaju et al. (2013) also reported a decrease in plasma T_3_ level in broilers kept under short/nonintermittent photoperiods compared to birds reared under long-continuous photoperiod, where T_3_ hormone is closely associated with feeding (McNabb, 2000). Thus, as observed in the present trial, light restriction can be used to restrict feed intake, with consequences on growth rate, myopathy occurrence and meat quality, besides behavior of broiler chickens.

Consistently with our results, previous studies also reported a decrease of feed intake when light hours were less than 18 without differences in FCR among the chickens kept at 24L:0D, 18L:6D, 8L:16D, and 4L:20D (Schwean-Lardner et al., 2016; Kim et al., 2022). Under our conditions, since all the chickens were kept under the same light regime until 9 d of age, the light restriction acted as a late feed restriction. In fact, Gratta et al. (2019) reported that early-feed-restricted chickens achieved the same final LW as chickens fed ad libitum due to a compensatory growth during the refeeding period, whereas late feed restriction resulted in a lower final LW compared to not restricted chickens. Trocino et al. (2015) reported a lower final LW in chickens submitted to early feed restriction compared to chickens fed ad libitum (−2%) despite the compensatory growth; on the other hand, the restricted animals showed a better FCR. In the present study, we observed a certain recovery in the performance of the light-restricted chickens during the last week of the trial when they exhibited a compensatory growth and were more active (lower rate of sitting/laying chickens) compared to the other group. In fact, previous studies (Classen et al., 1991; Sanotra et al., 2002; Classen, 2004) reported that bone metabolism and leg health improved and, thus, walking ability and activity increased in light-restricted broiler chickens. Other welfare benefits have been described in light-restricted chickens (8L:16D) compared to animals submitted to prolonged daylight, such as a decreased physiological stress and improved immune response (Classen, 2004; Olanrewaju et al., 2006).

As light restriction results in a lower feed intake, this could be effective for controlling the occurrence of myopathies as it happens for other metabolic disorders associated with the high growth rates of selected commercial genotypes (De Jong et al., 2012; Sahraei, 2012). In fact, Meloche et al. (2018) observed decreased scores for WB at 33 and 43 d of age, and for WS at 43 and 50 d when chickens were restricted at 95% of ad libitum intake, without additional reductions in myopathy scores with further reductions in feed intake. Simões et al. (2020) also reported that when feed restriction increased from 21 to 49 d of age, WB occurrence linearly decreased. Similarly, Toplu et al. (2021) found that feed restriction (70% of ad libitum intake between 11 and 24 d and 80 to 70% between 25 and 39 d of age) effectively reduced WS and WB occurrences and severity. On the other hand, under our conditions, differences in WB occurrence according to light restriction were not significant, likely because of the moderate sample size, whereas a significant reduction of total WS and severe WS breasts was measured and associated to reduced growth rate. Contrarily to previous studies, Gratta et al. (2019) found that neither early (13–23 d) nor late feeding restriction (27–37 d) affected WS or WB occurrence, whereas Trocino et al. (2015) reported that early feed restriction tended to increase the rate of WS breasts (69.5 vs. 79.5%; *P* < 0.10). These latter results depended on the higher growth rates of previously restricted chickens, associated to the compensatory growth during the realimentation period. In fact, at a histological level, Radaelli et al. (2017) found that early feed restriction reduced muscle fiber degeneration associated with WS and WB, but no residual effects were recorded at the end of the trial after a realimentation period during which chickens were fed ad libitum.

As for slaughter yield and carcass traits, these are dependent on the final LW of the chickens. Thus, Downs et al. (2006) reported greater leg proportions (+0.43%) in birds exposed to variable photoperiods (18L:6D until 35 d of age and then 23L:1D until 56 d) than in those receiving a constant photoperiod.

As for meat quality, the effect of the photoperiod on meat quality traits is little studied in broilers. The present results corroborate previous studies reporting no impact of photoperiod on pH, cooking, and thawing losses (Erdem et al., 2015; Fidan et al., 2017; Tuell et al., 2020; Kim et al., 2022), whereas effects on meat color are not consistent among studies (Fidan et al., 2017; Will et al., 2019; Tuell et al., 2020; Kim et al., 2022). The presents study also found an increase in meat redness with light restriction, consistently with Will et al. (2019) which recorded the lowest a* values in meat from birds exposed to the longest light period. Changes in meat color due to exposures to the different photoperiods have been associated with changes in meat oxidative stability and TBARS (Will et al., 2019). Then, consistently with our observations, Kim et al. (2022) measured higher shear force in the meat of birds kept under 24L compared to 8L:16D, whereas other authors (Will et al., 2019; Tuell et al., 2020) reported no impact of photoperiod on this quality trait.

Regarding chemical composition, increased growth rates have been associated with increased fat deposition (Tůmová and Teimouri, 2010). Namely, the increase in the light hours has been found to increase melatonin synthesis, inhibit the synthesis of insulin, and promote the synthesis of glucagon, favoring lipogenesis (Wang et al., 2020), which is consistent with the higher lipid meat content we found in chickens kept under the standard-light regime compared to those submitted to a shorter daylight period. In contrast, other authors (Tuell et al., 2020; Kim et al., 2022) did not report changes in meat chemical composition associated with the photoperiod.

### Effect of Genotype, Sex, and Age

Commercial production of broiler chickens is actually based on fast-growing high-breast genotypes, which guarantee favorable growth performance, carcass yield, and meat quality (Petracci et al., 2015; Maharjan et al., 2021) whereas several weak points have been identified for chicken welfare (EFSA AHAW Panel, 2023). According to Bailey et al. (2020), the heritability of myopathies is rather low (0.25 for WS and 0.07 for WB), whereas non-genetic factors are of greater importance in the development of defective meat. Thus, nor the present or previous studies (Trocino et al., 2015; Bordignon et al., 2022) found relevant differences in WS occurrence among fast-growing genotypes. On the other hand, as for WB in males, Bordignon et al. (2022) found that WB occurrence was significantly affected by genotype with large differences in the odds of having WB among 3 commercial genotypes. In addition, Bailey et al. (2015) reported that a commercial broiler line selected for high-breast yield had a greater occurrence of WB than another line selected for moderate-breast yield.

As for meat quality, large differences in meat quality are measured when comparing genotypes with different growth rates (slow vs. medium vs. fast) which reach slaughtering weight at different ages (Branciari et al., 2009). Thus, under our conditions, genotype had a minor effect on few rheological traits, like cooking losses and meat tenderness.

Importantly, differences in growth performance and body development between males and females are rather known and, consistently with our results, DWG, carcass yield, and drumstick yield are greater in male broilers, whereas breast and *P. major* proportions result higher in females (Baeza et al., 2010; Hristakieva et al., 2014; Santos et al., 2021). The occurrence of WS has been found to be similar in the 2 sexes in the present study as in previous ones (Kuttappan et al., 2013b; Bordignon et al., 2022). As for WB, previous studies and meta-analyses have demonstrated that WB occurrence is higher in males than in females (Brothers et al., 2019, Santos et al., 2021; Bordignon et al., 2022) despite the absence of significant differences observed in the present study. On the other hand, differences in meat quality between males and females were greater, significant, and consistent with previous studies. The higher meat pH in males is likely based on the strong negative correlation between breast weight and glycogen content (Bihan-Duval et al., 2008) where lower glycogen storage has been also related to myopathies occurrence (Abasht et al., 2016; Alnahhas et al., 2016). Additionally, a higher sarcoplasmic protein denaturation could explain the higher cooking losses we observed in males compared to females (Schneider et al., 2012). The higher cooking losses measured in meat from males likely accounted for the increased shear force measured on this meat compared to females, which is consistent with the findings of Fanatico et al. (2005). Since cooking losses are an indicator of the WHC, the lower protein content found in the breast of males could have resulted in a lower WHC, causing higher cooking losses and, therefore, meat more resistant to shear.

Importantly, differences in growth performance and body development between males and females and among different genotypes can affect animal behavior and welfare in terms of level of activity and diversification of behaviors. In the present study, the effect of the genotype was weak resulting in small differences in the rate of chickens that were observed feeding and drinking, which were consistent with differences in DFI, DWG, and final LW recorded between the 2 genotypes. As mentioned above, major differences according to genotypes are expected when comparing fast-growing with slow-growing genotypes where these latter have been found to exhibit a higher movement and a larger behavioral repertoire (Branciari et al., 2009).

On the other hand, despite differences in LW, under our conditions the behavioral pattern of the 2 sexes was rather similar. Previous papers found that a lower rate of walking males compared to females was probably a consequence of the higher LW of the former (McLean et al., 2002; Trocino et al., 2020). As for aggression, most of the studies focused on broiler breeders, being the males more aggressive than the females (Millman et al., 2000), whereas there is a scarcity of data on the aggressive behavior in broiler chickens. The studies exploring male-to-male interactions suggest that aggression arises mainly from competition for food in case of restriction (Mench, 1988) where high stocking density and group size can also favor aggressiveness in poultry species (Bilčík and Keeling, 2000; Estevez, 2007).

As for the effect of the age, findings about behavioral observations reported herein corroborate previous results describing a decline in activity (Bokkers and Koene, 2003) and feeding behavior (Bayram and Özkan, 2010; Trocino et al., 2020) over time in broiler chickens. The reduction of movement and the increase of inactive behaviors we observed with age have been attributed to the increased LW as well as the physical restriction of movement caused by both a shrinking in available floor space as bird size increases and a worsening of leg weakness and lesions developed over time (McLean et al., 2002). Moreover, in modern fast-growing high-breast genotypes, an increase in metabolic costs, associated with moving a heavy sternal mass during breathing and the consequent reduction in the respiratory capacity, can lead to behavioral changes such as an increased lay down and limited locomotion (Tickle et al., 2018). According to literature, some myopathies that develop over time, like WB, can also affect animal mobility by increasing locomotor difficulties (Norring et al., 2019) and disabling wing lifting due to the degenerative changes in the *P. major* muscles (Kawasaki et al., 2016).

As for the different behaviors, Mench (1988) found aggression to be of little significance in commercial broiler farming when birds were fed ad libitum, which is consistent with our results. As for specie-specific behaviors, dust bathing is as an important part of bird natural behavior in the function of balancing lipid levels in the feathers, improvement of feather structure, and removal of ectoparasites (Duncan, 1998; Sanotra and Weeks, 2004). In agreement with the present results, dust bathing activity has been reported to decrease with age due to the reduction in individual floor space as birds grow (Meluzzi and Sirri, 2009; Bayram and Özkan, 2010) or the deterioration in the litter quality over time (Shields et al., 2005).

## CONCLUSIONS

Under the condition of the present trial, a reduction of light hours depressed growth rate compared to a standard-lighting regime and was effective in decreasing WS occurrence and severity and in improving feed conversion. Moreover, the decrease in inactivity in light-restricted birds could be positively considered in view of chicken welfare. Finally, from a sustainability point of view, the light restriction, besides reducing WS occurrence and possibly wastes due to defective meat, could also reduce the energy and feeding costs.

## Appendix Supplementary materials

Image, application 1

## Footnotes

Supplementary material associated with this article can be found in the online version at doi:10.1016/j.psj.2023.103084.
