# Supplementary material for: Effect of light restriction on productive results and behavior of broiler chickens
Source: Poult Sci. 2023 Sep 1;102(12):103084. doi: 10.1016/j.psj.2023.103084 (PMC10568561; doi:10.1016/j.psj.2023.103084)
Supplement: Supplementary file 1 [file mmc1.docx]

**Effect of light restriction on behavior, performance and meat quality of broiler chickens**

F. Gratta^*^, M. Bošković Cabrol*, G. Xiccato^*^, M. Birolo^*^, F. Bordignon^*^, A. Trocino^*,#,1^

^*^Department of Agronomy, Food, Natural Resources, Animals and Environment (DAFNAE), University of Padova, Viale dell’Università 16, 35020 Legnaro, Padova, Italy. ^#^Department of Comparative Biomedicine and Food Science (BCA), University of Padova, Viale dell’Università 16, 35020 Legnaro, Padova, Italy.

**Table S1**. Significant interactions between genotype and sex (LS means) in broiler chickens from hatching until slaughtering at 45 d of age

|  |  | Genotype A | | Genotype B | |  | P value |
| --- | --- | --- | --- | --- | --- | --- | --- |
| Body weight (g)^1^ |  | Females | Males | Females | Males |  |  |
| Day 1 |  | 51.7^c^ | 51.5^c^ | 43.6^a^ | 47.6^b^ |  | <0.01 |
| Day 10 |  | 283^c^ | 286^c^ | 236^a^ | 275^b^ |  | <0.001 |
| Day 17 |  | 633^b^ | 666^c^ | 565^a^ | 659^c^ |  | <0.001 |
| Day 24 |  | 1136^b^ | 1248^c^ | 1027^a^ | 1221^c^ |  | <0.01 |
| Day 31 |  | 1745^b^ | 2027^d^ | 1589^a^ | 1971^c^ |  | <0.05 |
|  |  |  |  |  |  |  |  |
| Weight gain (g/d)^1^ |  |  |  |  |  |  |  |
| Days 1-10 |  | 25.7^b^ | 26.0^b^ | 21.3^a^ | 25.2^b^ |  | <0.001 |
| Days 10-17 |  | 49.9^b^ | 54.4^c^ | 47.0^a^ | 55.0^c^ |  | <0.01 |
|  |  |  |  |  |  |  |  |
| Feed intake (g/d)^2^ |  |  |  |  |  |  |  |
| Days 1-10 |  | 28.9^b^ | 29.6^b^ | 24.2^a^ | 27.9^b^ |  | <0.01 |
| Days 10-17 |  | 64.9^b^ | 67.5^bc^ | 60.6^a^ | 68.9^c^ |  | <0.01 |
| Days 17-24 |  | 105^b^ | 117^c^ | 93.0^a^ | 112^bc^ |  | <0.05 |

^1^Individual data; ^2^Pen data.

**Table S2.** *P*-value of the effect of interactions between photoperiod (P), genotype (G), sex (S) and age (A) on the behavior of broiler chickens.

|  |  | *P*-value | | | | | |  |
| --- | --- | --- | --- | --- | --- | --- | --- | --- |
|  |  | P×G | P×S | P×A | G×S | G×A | S×A | |
| Feeding |  | 0.67 | 0.73 | 0.99 | 0.09 | 0.74 | 0.71 | |
| Drinking |  | 0.13 | 0.70 | 0.48 | 0.11 | 0.62 | 0.13 | |
| Standing |  | 0.13 | 0.06 | <0.05 | 0.79 | 0.37 | 0.73 | |
| Sitting/lying |  | 0.13 | 0.23 | 0.06 | 0.22 | 0.50 | 0.25 | |
| Walking |  | 0.38 | 0.40 | <0.05 | 0.37 | <0.05 | 0.91 | |
| Pecking floor |  | 0.73 | 0.41 | <0.001 | 0.70 | 0.40 | <0.001 | |
| Pecking other birds |  | 0.08 | 0.97 | <0.001 | 0.50 | <0.001 | <0.001 | |
| Aggressiveness |  | 0.39 | 0.58 | 0.99 | 0.06 | 0.82 | 0.22 | |
| Pecking own tail |  | 0.08 | 0.22 | 0.14 | 0.91 | 0.72 | 0.89 | |
| Dustbathing |  | 0.84 | 0.20 | 0.72 | 0.13 | 0.61 | 0.98 | |
| Comfort |  | 0.92 | 0.68 | 0.30 | 0.46 | 0.31 | <0.05 | |

| 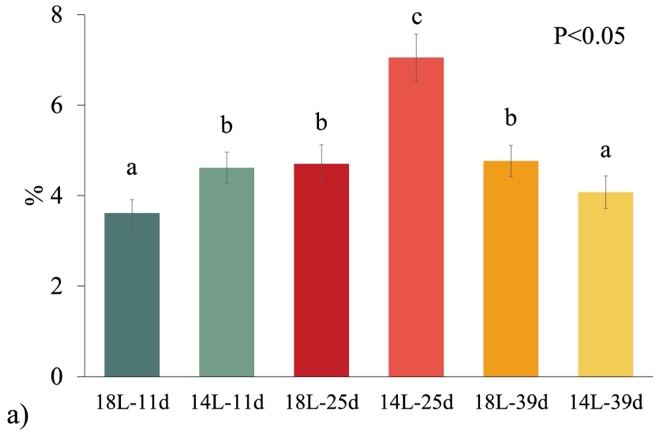 | 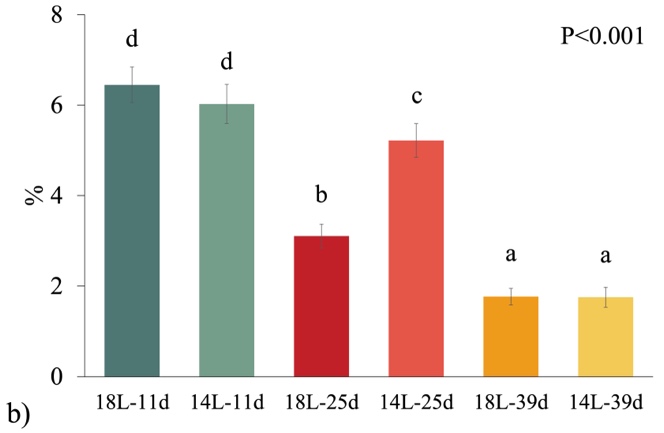 |
| --- | --- |
| 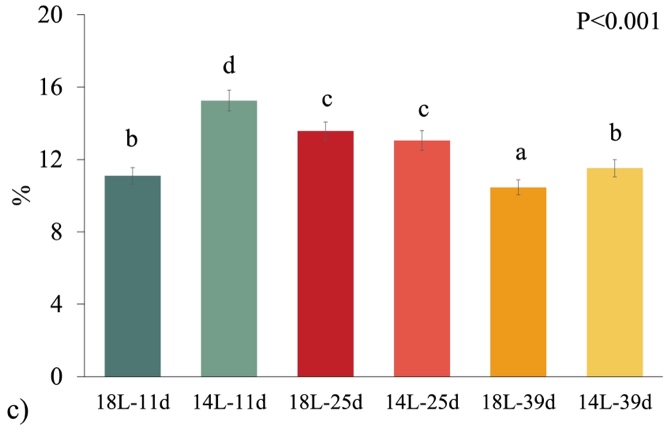 | 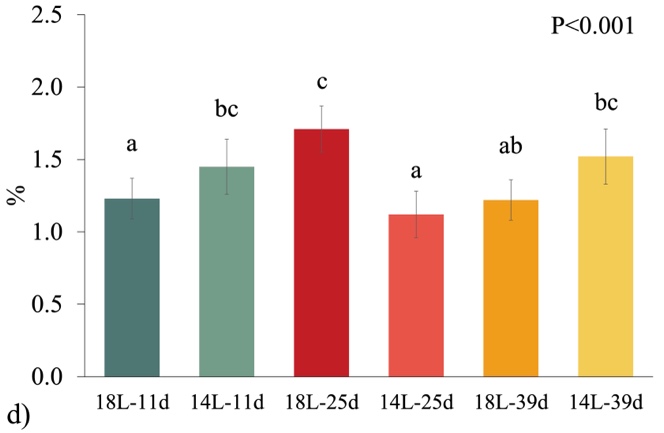 |

**Figure S1**. Effect of the interaction between photoperiod (18L:6D and 14L:10D) and age (11 d, 25 d and 39 d of age) on standing (a), walking (b), pecking floor (c) and pecking other birds (d) behaviors. Data are represented as means ± SEM.

| 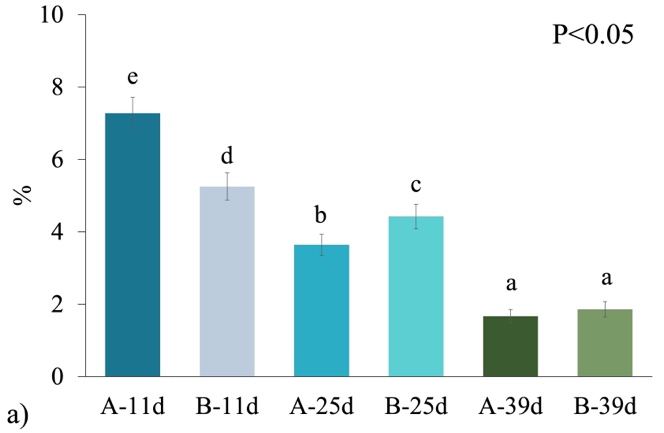 | 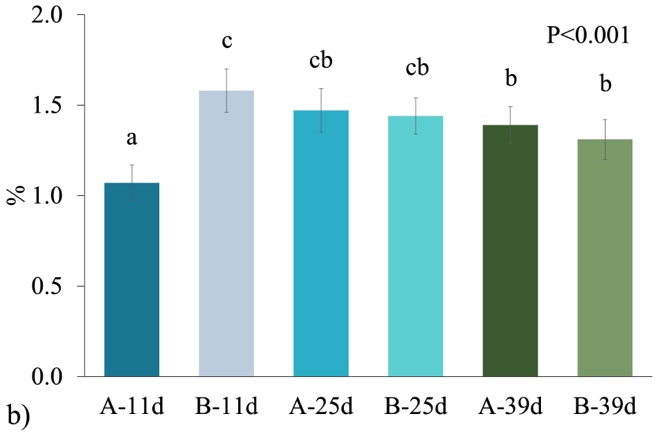 |
| --- | --- |

**Figure S2**. Effect of the interaction between genotype (A and B) and age (11 d, 25 d and 39 d of age) on walking (a) and pecking other birds (b) behaviors. Data are represented as means ± SEM.

| 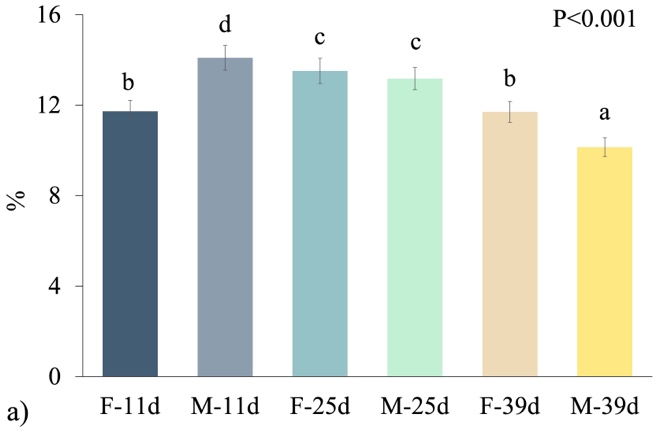 | 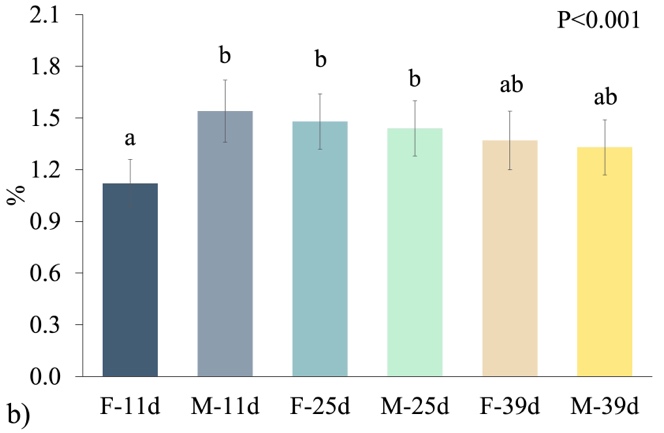 |
| --- | --- |
| 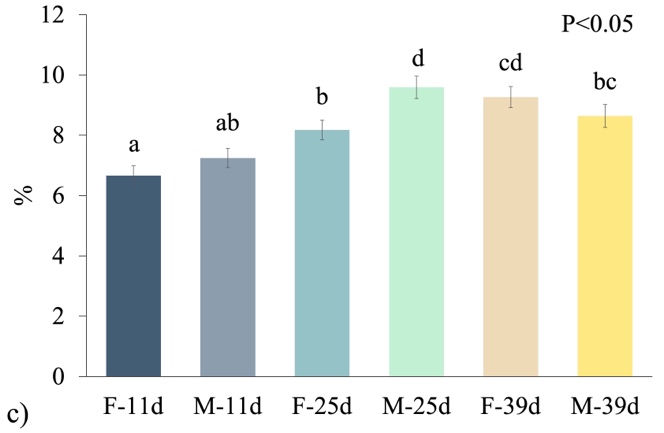 |  |

**Figure S3**. Effect of the interaction between sex (Female-F and Male-M) and age (11 d, 25 d and 39 d of age) on pecking floor (a), pecking other birds (b) and comfort (c) behaviors. Data are represented as means ± SEM.
